# Postpandemic fear of COVID‐19, psychological distress, and resilient coping among frontline health workers in Ghana: An analytical cross‐sectional study

**DOI:** 10.1002/hsr2.1608

**Published:** 2023-10-10

**Authors:** Evelyn Adu Fofie, Emmanuel Ekpor, Samuel Akyirem

**Affiliations:** ^1^ SDA Nursing and Midwifery Training College Asanta Ghana; ^2^ School of Nursing and Midwifery University of Ghana Legon Ghana; ^3^ School of Nursing Yale University New Haven Connecticut USA

**Keywords:** COVID‐19, fear, Ghana, psychological distress, resilient coping

## Abstract

**Background and Aims:**

The coronavirus disease 2019 (COVID‐19) pandemic has significantly impacted the psychological well‐being of healthcare workers globally. However, little is known about the mental health state of frontline health workers in the postpandemic era. The purpose of this study was to examine postpandemic COVID‐19‐related psychological distress and fear among frontline health workers in Ghana.

**Methods:**

Data were collected from 245 frontline healthcare workers in the Western region of Ghana. COVID‐19‐related fear, psychological distress, and resilient coping were assessed with the fear of COVID‐19 scale, Kessler Psychological Distress Scale, and the Brief Resilient Coping Scale, respectively. Multiple linear regression was used to assess the association between psychological distress and fear of COVID‐19 as well as the moderating effects of resilient coping.

**Results:**

Participants were mostly female (57.1%), aged between 21 and 30 years (50.6%), and not married (58.0%). We found that 52.2% of frontline healthcare workers experienced mild‐to‐severe fear, while 40% experienced psychological distress. Fear of COVID‐19, previous contact with COVID‐19 patients, and earning a monthly income between 501 and 1000 Ghanaian cedis were significantly associated with higher psychological distress. The positive association between fear of COVID‐19 and psychological distress was stronger among frontline health workers who had higher resilient coping.

**Conclusion:**

There is the need to provide frontline health workers with mental health support services to promote their psychological well‐being and enhance their ability to provide quality care during the postpandemic era.

## 1 INTRODUCTION

The coronavirus disease 2019 (COVID‐19) pandemic disrupted global economies and healthcare systems around the world. In Africa, about 9 million COVID‐19 cases were diagnosed and over 174,000 individuals died of the pandemic.^1^ In Ghana as of May 2023, a total of 171,657 cases and 1462 deaths had been recorded.^2^ Ghana's case prevalence and case fatality rates were among the lowest in the West African subregion.^3^, ^4^

At the height of the pandemic, healthcare systems worldwide were overwhelmed with new COVID‐19 cases.^5^ Health professionals experienced increased workload and were required to work overtime, adhere to dynamic infection prevention policies, and deal with constant exposure to traumatic situations.^6^ Previous studies have shown that the demands of the pandemic resulted in poor psychological outcomes among health professionals including increased psychological distress, burnout, depressive symptoms, anxiety, occupational stress, and posttraumatic stress disorder.^7^, ^8^, ^9^ For instance, a multicenter study in Ghana reported that 21.1% and 27.8% of frontline healthcare professionals had experienced depression and anxiety respectively, 5 months into the pandemic.^10^ A similar study conducted around the same time in a regional hospital in Ghana also showed a 71.1% prevalence rate of anxiety among health‐care professionals.^11^ These negative psychological outcomes among healthcare workers may have implications for patients' safety.^12^

For frontline healthcare workers in Ghana, the fear of COVID‐19 was real. As of June 2020, over 2000 healthcare workers in Ghana had been infected with COVID‐19.^13^, ^14^ The increased risk of getting infected, uncertainty about effective treatment and prevention modalities^15^ as well as the preponderance of COVID‐related stigma in Ghanaian communities^16^ may have fueled the fear of COVID‐19 among healthcare workers. Previous studies have shown a positive association between fear of COVID‐19 and poor psychological outcomes among different population groups in Ghana including university students^17^ and healthcare workers.^10^

Resilient coping refers to the use of cognitive appraisal skills to positively adapt to stressful situations.^18^ Resilient coping enables individuals to psychologically reframe the potency of a stressful event to reduce the impact of the stressor.^18^ Healthcare workers who have higher resilient coping tend to experience less perceived stress.^19^ Resilience coping is also known to moderate the effects of adverse stressors on psychological outcomes including depression, anxiety, and distress. Specifically, higher resilient coping reduces the impact of a stressor on psychological outcomes.^18^ Yet, it is not known if resilience coping can moderate the association between fear of COVID‐19 and psychological outcomes.

The World Health Organization officially declared COVID‐19 as no longer a global emergency on May 5, 2023.^20^ However, in Ghana, by late 2022, all COVID‐19 restrictions had been lifted and people were effectively living in the postpandemic era. Moreover, the number of new cases in Ghana had almost flattened out and the burden on healthcare professionals had significantly reduced by July–August 2022 in part due to the successes of vaccination efforts.^21^ It is, however, not known whether the fear of COVID‐19 and psychological distress among healthcare workers persist even in the postpandemic era. The purpose of this study was to examine postpandemic COVID‐19‐related psychological distress and fear among frontline health workers in Ghana. The study tests two hypotheses: (1) fear of COVID‐19 is associated with higher psychological distress; and (2) resilient coping decreases the effects of fear of COVID‐19 on psychological distress (effect modification).

## 2 METHODS

### 2.1 Study design

We used an analytical cross‐sectional study design to assess psychological distress and fear of COVID‐19 among frontline healthcare workers in Ghana during the postpandemic era. The study was conducted in three conveniently selected healthcare facilities in the Western Region of Ghana: St. Martin De Porres, Aiyinasi health Centre, and Esiama Health Centre. As of the time of participants' recruitment, there were 310 healthcare workers in these three sites. This study obtained Institutional Review Board (IRB) approval from the Ghana Health Service Ethics Review Committee (GHS‐ERC: 062/06/22). Furthermore, informed consent was obtained from each participant before data collection.

### 2.2 Study sample and data collection

Sample size was determined using an online sample calculator (https://sampsize.sourceforge.net/cgi-bin). Based on a previous study,^22^ we assumed that the prevalence of mild‐to‐severe psychological distress among frontline healthcare workers was 56.2%. In addition, we assumed a precision of 3% and a population size of 310. These parameters resulted in a sample size of 240 at 80% power. We conveniently sampled 245 frontline healthcare workers from the three clinical sites. We included persons who met the Centers for Disease Control and Prevention's Advisory Committee on Immunization Practices' (ACIP) definition of frontline health workers which includes all persons working in the healthcare settings who are likely to have direct or indirect contact with patients' or their bodily fluid.^23^ Thus, the inclusion criteria for this study were: being a medical officer, physician assistant, nurse, laboratory technician, emergency medicine technician (paramedic), hospital cleaner, or mortuary attendant who routinely come into direct contact with patients. We excluded healthcare professionals who were on leave at the time of data collection and those that were in administrative roles and had no contact with patients. Participants' recruitment and data collection occurred both online and in‐person from September to October 2022. An electronic questionnaire was designed and tested on several electronic devices to ensure that the questionnaire showed up correctly. A link to the electronic questionnaire was generated and shared on the official WhatsApp groups of each health facility with the help of the various WhatsApp group administrators. Trained research assistants from each of the three clinical sites helped to administer a paper questionnaire in‐person.

### 2.3 Measures

*Psychological distress* was measured using the 10‐item Kessler Psychological Distress Scale (KPDS).^24^ KPDS assesses stress levels and experiences of negative emotions on a 5‐point Likert‐type scale: *none of the time* (1), *a little of the time* (2), *some of the time* (3), *most of the time* (4), *and all of the time* (5). The total score is calculated by summing the individual scores. Higher scores on this scale indicate higher psychological distress. The scale is psychometrically validated.^25^ The Cronbach's *⍺* of the KPDS in this study was 0.88, indicating adequate internal consistency.

*Fear of COVID‐19* was measured using the 7‐item Fear of COVID‐19 Scale (FCS).^26^ The FCS was originally developed in Iran but has been validated in other countries including Egypt, Portugal, and Argentina.^27^, ^28^, ^29^ Each item on the FCS measures fear toward COVID‐19 infection on a 5‐point Likert‐type scale ranging from 1—*strongly disagree*, 2—*disagree*, 3—*neutral*, 4—*agree*, and 5—*strongly agree*. The total score ranges from 7 to 35, and it is obtained by summing up the score for each item on the scale. The higher the total score, the greater the fear of COVID‐19. In the present study, the FCS had good psychometric properties including a Cronbach's *⍺* of 0.83.

*Resilient coping* was measured using the 4‐item Brief Resilient Coping Scale (BRCS).^18^ The BRCS measures individuals' ability to adapt to or cope with stressful situations. The BRCS has good test–retest and internal reliability and has demonstrated acceptable convergent validity.^18^ The items on the instrument are scored on a 5‐point Likert‐type scale from 1 to 5 where 1 means “the statement does not describe me at all” and 5 means “the statement describes me very well.” Total resilient coping scores are obtained by adding up the scores of each item. Higher total scores indicate higher resilient coping. The Cronbach's *⍺* for the BRCS in this study was 0.69.

*Sociodemographic characteristics* included age ( < 21/21–30/31–40/41–50), gender (Male/Female), religion (Christian/Islam/Others), marital status (Married/Not married), level of education (up to a 3‐year Diploma/Bachelor's degree or higher), employment type (permanent/temporary), monthly income ( < 500/500–1000/1001–3000), working hours per week (up to 35 h/>35 h), working years, and presence of chronic illness (yes/no).

### 2.4 Data analysis

Data analysis was performed with R statistical software version 4.3. Descriptive statistics (mean, median, standard deviations, frequencies, and proportions) were used to summarize psychological distress, fear of COVID‐19, resilient coping, and sociodemographic characteristics. Secondly, to determine the prevalence of psychological distress, we further dichotomized the KPDS into no distress and mild‐to‐severe distress, using a cut‐off total KPDS score of < 20 and ≥ 20, respectively.^30^ We also computed the prevalence of mild‐to‐extreme fear of COVID‐19 by dichotomizing scores into no fear (7–21) and mild‐to‐extreme fear (22–35). Third, multiple linear regression was performed with psychological distress as outcome and fear of COVID‐19 as predictor while adjusting for sociodemographic characteristics. We assessed the variance inflation factor (VIF) of all independent variables to ensure all VIF were less than 4 to prevent collinearity. We natural log‐transformed the total KPDS score to ensure the assumption of normality was met. To identify a parsimonious model, we repeated the linear regression, but with stepwise selection. The Alkaike Information Criteria (AIC) was used to select the best model. We also assessed the moderating effect of resilient coping by adding it to the final parsimonious regression model as an interaction term. *p* value less than or equal to 5% (two‐sided significance level) was considered statistically significant.

## 3 FINDINGS

Table 1 shows descriptive statistics of the study sample. Overall, most participants were female (57.1%), aged between 21 and 30 years (50.6%), not married (58.0%), and had up to a 3‐year diploma certificate (79.6%). Majority of participants had no chronic disease (94.7%) and were early in their career, having worked for only 1–3 years (38.8%). Only 40% of the frontline health workers reported experiencing mild‐to‐severe psychological distress. The prevalence of mild‐to‐extreme fear of COVID‐19 was 52.2%. The mean (standard deviation) FCS and BRCS scores were 21.5 (6.2) and 16.5 (3.0), respectively.

**Table 1:** Characteristics of study sample.

Characteristics	*N* (%)
Age	
<21 years	15 (6.1)
21–30 years	124 (50.6)
31–40 years	87 (35.5)
41–50 years	19 (7.8)
Gender	
Male	105 (42.9)
Female	140 (57.1)
Marital status	
Married	103 (42.0)
Not married	142 (58.0)
Religion	
Christian	198 (80.8)
Islamic	35 (14.3)
Traditional/Others	12 (4.9)
Education	
Up to 3‐year diploma	195 (79.6)
University degree or higher	50 (20.4)
Employment type	
Permanent	178 (72.7)
Temporary	67 (27.3)
Monthly income	
≤500 GHC	65 (26.5)
501–1000 GHC	67 (27.3)
1001–3000 GHC	95 (38.8)
>3000 GHC	18 (7.3)
Chronic disease	
No	232 (94.7)
Yes	13 (5.3)
Working hours/week	
Up to 35 h	197 (80.4)
>35 h	48 (19.6)
Direct contact with COVID‐19 patients	
No	134 (54.7)
Yes	111 (45.3)
Years of work	
Less than a year	51 (20.8)
1–3 years	95 (38.8)
4–7 years	53 (21.6)
8–15 years	35 (14.3)
15+ years	11 (4.5)
KPDS	
Median (IQR)	18 (14‐23)
FCS	
Mean (SD)	21.5 (6.2)
BRCS	
Mean (SD)	16.5 (3.0)

Abbreviations: BRCS, Brief Resilient Coping Scale; COVID‐19, coronavirus disease 2019; FCS, Fear of COVID‐19 Scale; GHC, Ghanaian Cedis (1 US dollar = 13 GHC at the time of data collection); IQR, Interquartile Range; KPDS, Kessler Psychological Distress Scale; SD, standard deviation.

### 3.1 Association between fear of COVID‐19 and psychological distress

Table 2 shows the results of the two multiple linear regression models. In the full regression model, fear of COVID‐19 (*β* = 0.012, *p* = 0.001), previous contact with COVID‐19 patients (*β* = 0.104, *p* = 0.023), and earning a monthly income between 501 and 1000 Ghanaian cedis (*β* = 0.164, *p* = 0.015) were all significantly associated with higher psychological distress. In contrast, compared with being single, being married was significantly associated with lower psychological distress among frontline health workers in Ghana (*β* = −0.105, *p* = 0.044). We did not observe any significant association with religious affiliation, level of education, number of hours worked per week and the presence of chronic illness (*p* > 0.05). Similar results were observed in the parsimonious model with fear of COVID‐19, previous contact with COVID‐19 patients and a monthly income between 501 and 1000 Ghanaian cedis being significantly associated with higher psychological distress. Although the number of working hours was retained in the parsimonious model, this variable did not reach statistical significance. The parsimonious model was slightly better (Adjusted *R* ^2^: 12.3%) than the full model (Adjusted *R* ^2^ 11.9%) in explaining the variance in psychological distress. The VIFs of all independent variables included in the full model were all less than four indicating the absence of multicollinearity.

**Table 2:** Factors associated with psychological distress among frontline health workers.

	Full model		Parsimonious model	
Variables	Coefficient (β)	*p* value	Coefficient (β)	*p* value
FCS	0.012	0.001	0.011	0.001
Marital status				
Not married	Reference		Reference	
Married	–0.105	0.044	–0.125	0.008
Monthly income				
Up to 500 GHC	Reference		Reference	
501–1000 GHC	0.164	0.015	0.168	0.005
1001–3000 GHC	–0.027	0.693	0.007	0.904
>3000 GHC	0.099	0.373	0.106	0.275
Direct contact with COVID‐19 patients				
No	Reference		Reference	
Yes	0.105	0.023	0.103	0.023
Working hours				
Up to 35 h	Reference		Reference	
>35 h	–0.093	0.108	–0.095	0.089
Chronic illness				
No	Reference		Reference	
Yes	–0.108	0.285	–0.136	0.159
BRCS	–0.007	0.362		
Employment type				
Temporary	Reference		‐	‐
Permanent	0.051	0.419	‐	‐
Working years				
Less than a year	Reference		‐	‐
1–3 years	–0.022	0.744	‐	‐
4–7 years	–0.062	0.405	‐	‐
8–15 years	–0.030	0.722	‐	‐
15+ years	–0.229	0.073	‐	‐
Chronic illness				
No	Reference		‐	‐
Yes	–0.108		‐	‐
Sex				
Male	Reference		‐	‐
Female	–0.045	0.317	‐	‐
Education				
Up to diploma	Reference		‐	‐
Degree or higher	0.052	0.416	‐	‐
Religion				
Christian	Reference		‐	‐
Muslim	–0.002	0.974	‐	‐
Traditional/others	0.134	0.201	‐	‐
Model fit	Adjusted *R* ^2^: 11.9%, *F*(18,226) = 2.827, *p* = 0.0002		Adjusted *R* ^2^: 12.3%, *F*(8,236) = 5.289, *p* < 0.0001	

Abbreviations: BRCS, Brief Resilient Coping Scale; FCS, Fear of COVID‐19 Scale; GHC, Ghanaian Cedis (1 US dollar = 13 GHC at the time of data collection).

### 3.2 Moderation effects of resilient coping

**Figure 1:**
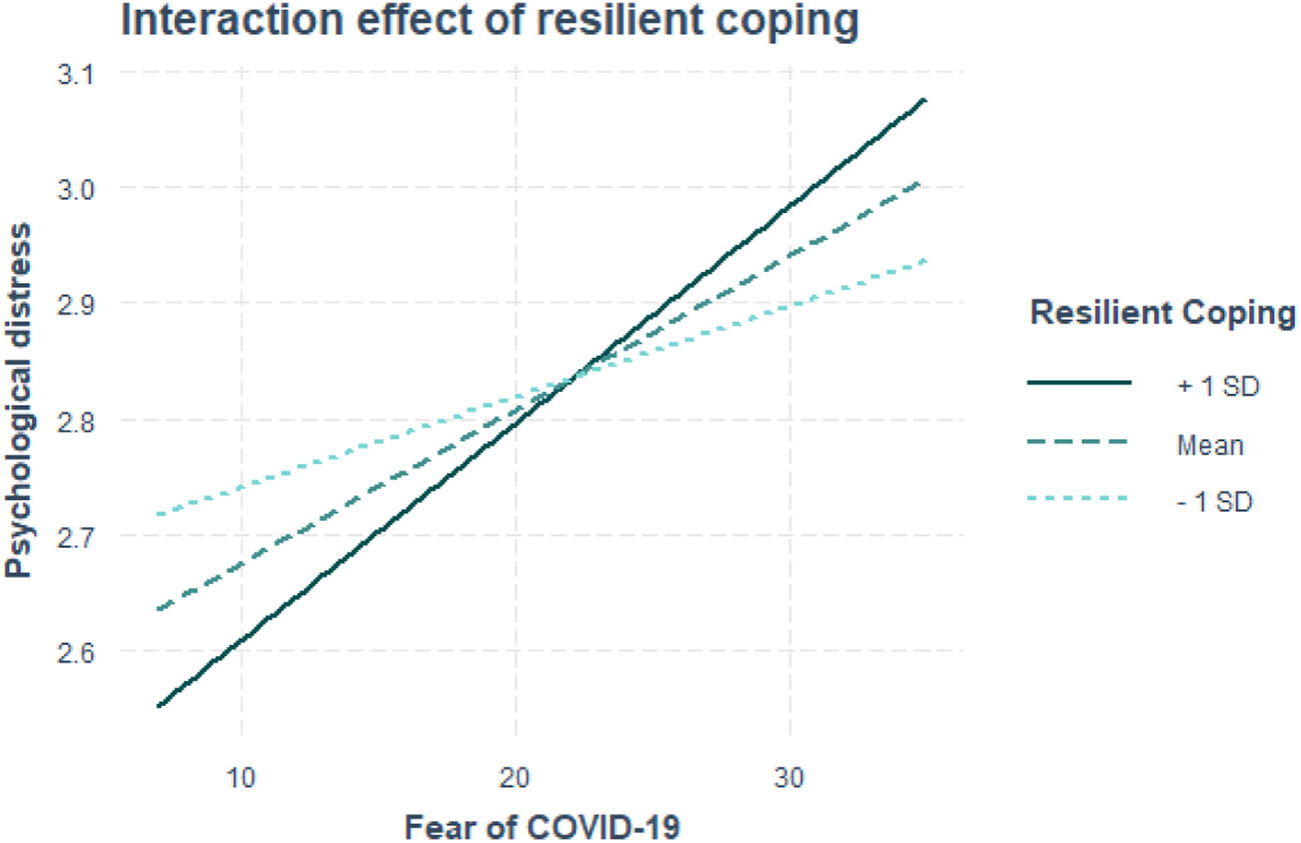
Interaction plot.

**Figure 2:**
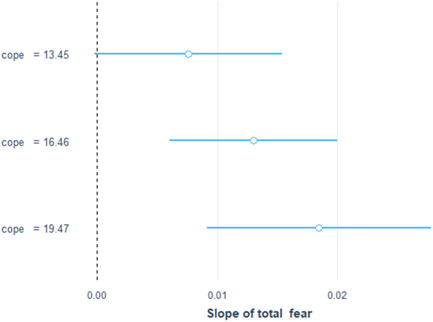
Effects of fear of coronavirus disease 2019 (COVID‐19) on psychological distress at different Brief Resilient Coping Scale (BRCS) scores.

When *resilient coping* × *fear of COVID‐19* interaction term was added to the parsimonious model, we found no significant association between fear of COVID‐19 and psychological distress. Resilient coping was found to significantly moderate the association between fear of COVID‐19 and psychological distress. Specifically, the positive association between fear of COVID‐19 and psychological distress was stronger among frontline health workers who had higher resilient coping. Further details are provided in Table 3 and Figures 1 and 2.

**Table 3:** Linear regression model with interaction term.

Variable	Coefficient (β)	*p* value
FCS	–0.017	0.228
BRCS	–0.040	0.020
Marital status		
Not married	Reference	
Married	–0.118	0.012
Income		
Up to 500 GHC	Reference	
501–1000 GHC	0.185	0.002
1001–3000 GHC	0.014	0.806
>3000 GHC	0.110	0.255
Direct contact with COVID‐19 patients		
No	Reference	
Yes	0.100	0.025
Working hours		
Up to 35 h	Reference	
>35 h	–0.088	0.117
Chronic illness		
No	Reference	
Yes	–0.143	0.136
FCS × BRCS	0.002	0.033

Abbreviations: FCS, Fear of COVID‐19 Scale; BRCS, Brief Resilient Coping Scale; GHC, Ghanaian Cedis (1 US dollar = 13 GHC at the time of data collection).

## 4 DISCUSSION

This cross‐sectional study aimed at examining the mental health state of frontline health workers in Ghana during the postpandemic era. The findings from this study reveal that even in the postpandemic era, healthcare workers in Ghana continue to experience poor psychological well‐being. This study is also the first to examine the moderating effects of resilience coping on psychological distress among frontline health workers in Ghana.

The current study revealed concerning findings regarding the psychological well‐being of frontline healthcare workers. Shockingly, a significant 52.2% of these dedicated health workers reported experiencing mild‐to‐severe COVID‐19 fear. This figure surpasses the 45.5% prevalence of fear among healthcare workers in Ghana, as documented in a prior study conducted at the height of the pandemic in 2020^10^ and a 19% prevalence rate in a study of healthcare workers across 14 countries.^31^ Also, 45.4% disclosed experiencing mild‐to‐severe psychological distress which is very alarming. Despite the decline in COVID‐19 cases in Ghana,^1^ it is disconcerting to observe heightened levels of fear and distress among healthcare providers in this postpandemic era. This clearly indicates that healthcare workers may be grappling with the lingering effects of the intense stress and anxiety they experienced during the peak of the crisis. These findings align with a previous longitudinal study,^32^ which revealed worsened symptoms of psychological stress among frontline workers during a 1‐year follow‐up period.

The relationship between fear and psychological distress, particularly in the context of COVID‐19, has indeed been extensively studied.^31^, ^33^, ^34^ Research has consistently shown that fear is a significant precursor for psychological distress, and this association has been observed in various populations, including healthcare workers. For instance, Ghozy et al. in their cross‐sectional analyses from 14 countries reported that healthcare workers who experienced higher levels of fear related to COVID‐19 also exhibited higher levels of psychological distress.^31^ Likewise, we also found that healthcare providers who reported fear of COVID‐19 were more associated with psychological distress. The COVID‐19 pandemic has introduced numerous stressors and uncertainties, leading to heightened fear and anxiety particularly among frontline health workers. This can trigger physiological and psychological responses that can manifest as symptoms of distress. Furthermore, we found that healthcare workers who had previous contact with COVID‐19 patients were more prone to experiencing psychological distress. This finding aligns with a similar study conducted in Ecuador.^35^ These significant findings underscore the impact of fear and exposure to COVID‐19 on the psychological well‐being of healthcare workers in Ghana. Recognizing and addressing the psychological well‐being of healthcare professionals is crucial, as their mental health directly influences their ability to deliver quality care to patients.

While fear can contribute to psychological distress, studies have shown that resilient coping may help people to reduce the negative impact of fear of COVID‐19 on their mental health by enhancing their sense of control and optimism.^36^ The moderating role of resilient coping in mitigating the association between fear and psychological distress has been established in various studies using statistical models. Elsayed et al. in their cross‐sectional study found that BRCS score showed an inverse relationship with the distress and fear scores, indicating that people with better coping had lower distress and fear of COVID‐19.^37^ Similar result was reported in a study conducted on healthcare workers in Italy.^38^ Conversely, we found that the positive association between fear of COVID‐19 and psychological distress was even stronger among frontline health workers who had higher resilient coping. These contrasting results may indicate that while resilience coping strategies are generally beneficial for reducing fear and psychological distress, it is possible that in some cases, they may inadvertently increase fear and distress. It is likely that the unique circumstances and challenges faced by frontline health workers in Ghana, such as limited resources and increased workload, may have influenced their psychological well‐being differently compared with other populations.^39^

This study has implications for practice and research. The study highlights the need to provide frontline health workers with mental health support services to promote their psychological well‐being and enhance their ability to provide quality care during the postpandemic era. Future studies should adopt a qualitative approach to tease out the factors that continue to drive COVID‐19 fear and psychological distress among healthcare workers. Future studies should also focus on evaluating the effectiveness and reach of mental health support services in the Ghanaian health system to help strengthen the evidence base and address implementation challenges of these services.

### 4.1 Strength and limitations

This study had several limitations. First, the cross‐sectional design does not allow us to infer causality, limiting our ability to make definitive conclusions regarding the pandemic's effects on psychological well‐being. Second, we did not collect data on the specific profession of participants (e.g., nurse, doctor). Such data would have allowed for granular analysis to determine which healthcare professionals experience the highest fear and psychological distress. Third, the use of subjective self‐reported measures could have introduced social desirability bias in this study. Fourth, this study was limited to only one of the 16 administrative regions of Ghana. Hence, our ability to make generalization is limited. Fifth, the use of convenience sampling may have resulted in a biased sample that does not adequately reflect the whole population of frontline healthcare workers in Ghana, potentially weakening the findings' generalizability. Despite these limitations, this study is the first to examine the role of resilient coping on psychological distress among frontline health workers in Ghana. It is also the first to examine their mental health state of health workers in Ghana following the end of the COVID‐19 pandemic.

## 5 CONCLUSION

We found evidence of poor psychological well‐being following the end of the COVID‐19 pandemic in Ghana. The evaluation of the moderating effects of resilient coping in this study is formative. Further studies using larger, diverse, and representative sample are required to confirm the paradoxical role of resilient coping. The findings from this study highlight the need to provide frontline health workers with mental health support services to promote their psychological wellbeing and enhance their ability to provide quality care during the postpandemic era.

## AUTHOR CONTRIBUTIONS

**Evelyn Adu Fofie**: Conceptualization; data curation; investigation; methodology; writing—original draft; writing—review & editing. **Emmanuel Ekpor**: Data curation; formal analysis; investigation; writing—original draft; writing—review & editing. **Samuel Akyirem**: Formal analysis; investigation; methodology; supervision; visualization; writing—original draft; writing—review & editing.

## CONFLICT OF INTEREST STATEMENT

The authors declare no conflict of interest.

## ETHICS STATEMENT

We obtained Institutional Review Board (IRB) approval from the Ghana Health Service Ethics Review Committee (GHS‐ERC: 062/06/22). Furthermore, informed consent was obtained from each participant before data collection.

## TRANSPARENCY STATEMENT

The lead author Emmanuel Ekpor affirms that this manuscript is an honest, accurate, and transparent account of the study being reported; that no important aspects of the study have been omitted; and that any discrepancies from the study as planned (and, if relevant, registered) have been explained.

## Data Availability

The data that support the findings of this study are available from the corresponding author upon reasonable request.
